# *In vitro* expression of cytokeratin 18, 19 and tube formation of adipose‐derived stem cells induced by the breast epithelial cell line HBL‐100

**DOI:** 10.1111/jcmm.12673

**Published:** 2015-09-28

**Authors:** Jie Yang, Lingyun Xiong, Rongrong Wang, Quan Yuan, Yun Xia, Jiaming Sun, Raymund E. Horch

**Affiliations:** ^1^ Department of Plastic and Reconstructive SurgeryUnion HospitalHuazhong Science & Technology UniversityWuhanHubeiChina; ^2^ Department of Plastic and Hand Surgery and Laboratory for Tissue Engineering and Regenerative MedicineUniversity Hospital ErlangenFriedrich Alexander UniversityErlangen‐NuernbergFAUGermany

**Keywords:** ADSCs, cytokeratin 18, cytokeratin 19, HBL‐100

## Abstract

Fat transplantation is increasingly used in breast augmentation; and recently, the issue of safety concerns from a cellular and molecular point of view has been raised. In this study, attentions were paid to the interaction between adipose‐derived stem cells (ADSC) and mammary epithelial cells: human breast cancer cell line ‐ 100 (HBL ‐ 100) cells were used to simulate the normal microenvironment in breast tissue, ADSCs were harvest from human and co‐cultured with HBL‐100 cells. It was found that ADSCs formed tube‐like structures in the co‐culture with HBL‐100 cells in contrast to the normal morphology of ADSCs in the control group. In addition, the immunofluorescence imaging showed that cytokeratin 18 and 19 (CK18 and 19) were significantly expressed in ADSCs after the co‐culture with HBL‐100 cells. The ultrastructure of those ADSCs also showed epithelial changes. In conclusion, ADSCs are not biological stable when co‐cultured with HBL‐100 cells. They differentiate into epithelial‐like cells with the expression of epithelial surface marks (CK 18, 19) and form tube‐like structures. This may offer an important evidence for the further study of clinical application of transplanting ADSCs rich adipose tissue into the breast in the future.

## Introduction

Aspirated human fat is a rich source of adipose‐derived stem cells (ADSC) for a multitude of applications in research and medicine 1, 2. Fat grafting has therefore gained increasing attention and has also been widely used for volume restoration in cosmetic and reconstructive surgery, including breast enlargement 3. However, even with the best surgical technique, the long‐term survival rate of fat grafts varies greatly. To overcome this limitation, a large number of studies have been performed to enrich fat grafts with various ingredients, like platelet‐rich plasma, VEGF, insulin‐like growth factor‐1, basic fibroblast growth factor, epithelial growth factor, stromal vasular fraction (SVF), etc. 3. Likewise, Yoshimura *et al*. supplemented fat grafts with SVF for purely cosmetic breast augmentation and reported excellent cosmetic outcome without any major complications 4. However, consistent long‐term clinical outcome so far are not yet available. Therefore, the issue of safety concerns from a cellular and molecular point of view has been raised when treating breast tissue in patients with a breast cancer history or in young females.

During breast cancer progression, dynamic communication between epithelial and stromal compartments occurs with a large panel of cytokines, chemokines and growth factors, which are essential for the generation of a more favourable microenvironment for tumour growth. Similarly, fat grafting in proximity to the mammary gland tissue might change the microenvironment of the breast by the addition of multipotent stem cells or other stimulating factors.

It has been shown that mammary gland macrophages play a key role in supporting stem/progenitor cell function and hence it was suggested that mammary stem cells require macrophage‐derived factors to be fully functional. Macrophages which are activated by ADSC may therefore constitute part of the mammary stem cell niche 5. Further on, the resident mammary epithelial cells might in turn also influence the grafted adipose‐derived stem cells. Therefore, currently it cannot be assured that fat grafting to the human breast does neither induce new cancer cells nor stimulate dormant cancer cells, as has been shown experimentally 5, 6, 7. In addition the biological behaviour of grafted ADSCs in normal breast tissue is nearly unknown. This study aims at investigating the potential relation and effects of ADSC on mammary epithelia.

HBL‐100, a human breast epithelial cell line, was obtained from primary cultures of cells derived from an early lactation sample of human milk. It is commonly used in breast related researches to simulate the normal microenvironment in breast tissue. In the preliminary study, influences of HBL‐100 on ADSCs were evaluated in an indirect co‐culture system. Adipose‐derived stem cells were isolated from lipoaspirate and co‐cultured with HBL‐100 cell line. Changes of cellular morphology of ADSCs were observed, and expression of epithelial surface makers of ADSCs was determined by immunofluorescence analysis. Further, ultrastructures of ADSCs were examined by high‐resolution transmission electron microscopy.

## Materials and methods

The experiment was approved by the ethics board of Union Hospital (Wuhan, China) and conducted in accordance with the Declaration of Helsinki. The HBL‐100 cell line (CX0129HBL‐100480) was purchased from Boster Biotech (Wuhan, China).

### Isolation of ADSCs

Seven female patients were included in the present study, the including criteria were as follows: no primary disease, age between 18 and 40 years, no liposuction history. Adipose‐derived stem cells were harvested from the adipose tissue of seven female patients undergoing liposuction between July 2011 and April 2012. The isolation was performed according to our previous experiences 8, 9. After digestion with collagenase I (Invitrogen, Carlsbad, CA, USA), centrifugation and filtration, The supernatant (adipocytes, oil) was discarded, the obtained cell fractions were cultured with DMEM/F12 (Hyclone; Thermo, Logan, UT, USA) supplemented with 10% foetal bovine serum (FBS; Gibco, Carlsbad, CA, USA), 100 U/ml of penicillin, and 100 mg/ml of streptomycin (Hyclone; Thermo) at 37°C in the presence of 5% CO_2_. Forty‐eight hours later, the non‐adherent fractions were removed and the remaining cells were washed with PBS (Hyclone; Thermo). The medium was replaced every 3 days thereafter. When 80% confluence was reached, the cells were digested and reseeded into T25 flasks (Corning, New York, NY, USA) at ratio of 1:2.

Adipose‐derived stem cells were successfully isolated from all seven adipose tissue samples. Unfortunately two samples of ADSCs were lost during cell culture because of contaminate. Therefore, the following experiments were carried out using the rest five samples.

### Co‐culture of ADSCs and HBL‐100 cells

Adipose‐derived stem cells (3rd passage) and HBL‐100 cells were synchronized by culturing 24 hrs in DMEM/F12 supplemented with 100 U/ml of penicillin and 100 mg/ml of streptomycin (Hyclone; Thermo) at 37°C and 5% CO_2_.

After the incubation without FBS for 24 hrs, the co‐culture system was constructed as follows: For the experimental group (*n* = 5), ADSCs and HBL‐100 cells were seeded in lower and upper chambers at a density of 1 × 10^5^/well, respectively. For the control group (*n* = 5), ADSCs were seeded in both lower and upper chambers at a density of 1 × 10^5^/well, and 24‐well Transwell plates (Corning) with 8‐μm pore membranes and glass coverslips in lower chambers were used in the transwell co‐culture.

Both experimental and control group were then incubated with DMEM/F12 supplemented with 10% foetal bovine serum, 100 U/ml of penicillin, and 100 mg/ml of streptomycin at 37°C and 5% CO_2_. The medium was replaced every 3 days thereafter. Cell morphologies of both groups were observed with an inverted microscope daily (Olympus, Tokyo, Japan).

### Immunofluorescence analysis

On the 15th day of the co‐culture, ADSCs of both groups were fixed with 4% paraformaldehyde for 2 min. and processed with 0.5% Triton X‐100 for 10 min. After washing with PBS, the cells were blocked in 5% bovine serum albumin (Hyclone; Thermo) for 1 hr at 24°C. Then the cells were washed and incubated at 4°C with mouse anti‐human antibodies against human cytokeratin (CK) 18 and 19 (Gene Tex, Irvine, CA, USA) in a ratio of 1:100 at 4°C. Twelve hours later, the cells were incubated with fluorescence labelled rabbit antimouse antibodies in a ratio of 1:100 (FITC or Texas Red‐conjugated; Beckton Dickinson, Franklin Lakes, NJ, USA) at 24°C for 10 min. After washing with PBS, cells were counterstained with 4′,6′‐diamidino‐2‐phenylindole (Sigma‐Aldrich, St. Louis, MO, USA) for 1 min. The images were acquired with a fluorescence microscope (Olympus).

### Ultrastructural observation

On the 15th day of the co‐culture, ADSCs were harvested and centrifuged, and then were fixed in 2.5% glutaric dialdehyde (Boster Biotech). After incubation at 4°C for 12 hrs, the cells were fixed in 1% osmic acid (Boster Biotech) at 4°C for 1 hr, dehydrated in graded acetone series, and embedded in Epon 812 (Boster Biotech). Ultrastructural identification was performed under Tecnai G220TWIN transmission electron microscope (Fei, Hillsboro, OR, USA).

### Statistical analysis

Positive rates of CK 18, 19 expressions in ADSCs were obtained as follows: 5 fields per sample were chosen randomly under a fluorescence microscope at 200× magnification. The positive rate was the number of CK 18, 19 positive cells dividing by the number of all cells.

Statistical analyses were performed with SPSS 13.0 (SPSS Inc., Chicago, IL, USA). Positive rates were analysed by Student's *t*‐test. All statistical analyses were two‐sided end, *P* < 0.05 was considered significant.

## Results

### Tube formation

**Figure 1:**
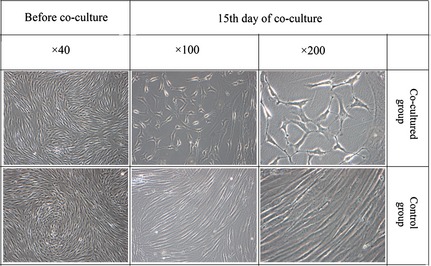
Morphologies of both groups. Tube formation was observed on the 15th day of co‐culture. There was no significant change in cellular morphology in control group.

Adipose‐derived stem cells formed tube‐like structures in the co‐culture with HBL‐100 cells in contrast to the normal morphology of ADSCs in the control group. After co‐culture for 15 days, the size of tube‐like structures increased significantly, the morphological differences between ADSCs in experimental groups and HBL‐100 cells were not obvious (Fig. 1). In addition, as early as 7 days of co‐culture, the long‐axis of ADCSs begun to decrease, pseudopodium‐like structures have been observed.

### Expressions of CK 18 and 19

**Figure 2:**
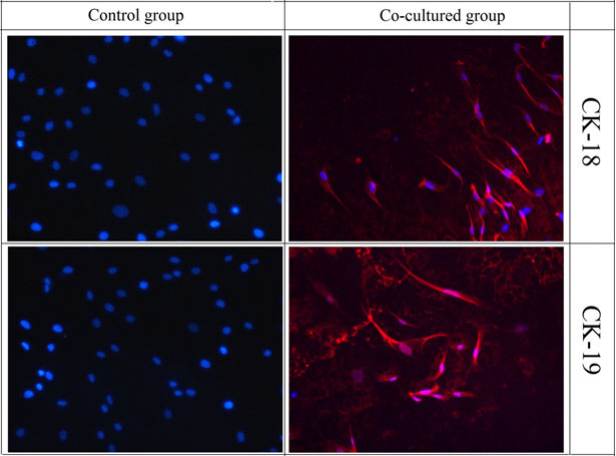
Expression of CK 18, 19 in co‐cultured group. No significant expression was observed in control group.

The immunofluorescence imaging showed that CK 18 and 19 were significantly expressed in the experimental group; in addition, no obvious expression of CK 18 and 19 were observed in control groups (*P* < 0.05, Fig. 2).

### Ultra‐structural alterations

**Figure 3:**
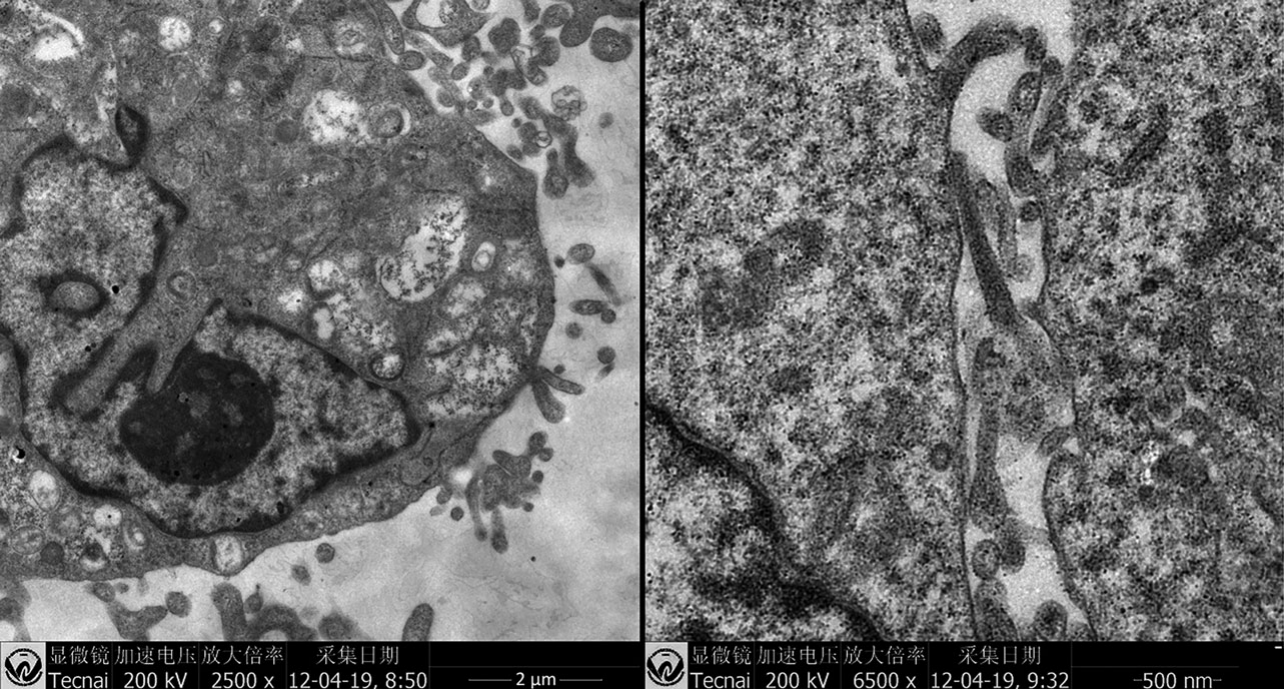
The ultrastructure of adipose‐derived stem cells (ADSCs) in co‐cultured group (left). Desmosomes were presented between cells (right).

The ultrastructure of ADSCs from experiment group showed epithelioid changes. After co‐culture, the nuclei were round and clear, and the chromatin inside each nucleus was homogeneous. Tonofilaments were increased around the nuclei. Desmosomes were presented between cells. No significant change was observed in control group (Fig. 3).

## Discussion

This study suggests that ADSCs could differentiate into epithelial‐like cells in the microenvironment of HBL‐100 cells. Adipose‐derived stem cells co‐cultured with HBL‐100 cells present typical epithelial properties, such as tube formation and expression of epithelial surface markers (CK 18, 19), which are distinct from ADSCs in the absence of HBL‐100 cells 10.

Adipose‐derived stem cells are reported to improve the survival rate of fat grafts. However, opinions on long‐term safety of transplanted ADSCs in breasts are controversial despite that cell‐assisted lipotransfer becomes increasingly popular in both purely cosmetic and reconstructive breast enlargement surgeries 11, 12, 13, 14, 15. In addition to the secretions of cytokines, the biological stability of ADSCs after transplantation would be another key factor which determines their clinical safety, considering the high incidence of breast carcinoma 16 as well as the multilayer injection technique applied during fat transfer 13.

In this study, we observed that ADSCs formed tube‐like structures as well as expressing CK 18 and CK19 after the co‐culture with HBL‐100. Previously, it has been reported that mesenchymal stem cells can be differentiate into endothelial cells during the angiogenesis of tumours and tube formation was detected 15, 17, 18, 19, 20. In our research, the transwell co‐culture was used to create a microenviroment in which two kinds of cells could interact with each other. In addition to those previously existing evidence 20, our results specifically indicated that ADSCs can be differentiate into epithelial‐like cells under the impact of the HBL‐100 cells. We supposed that ADSCs formed mammary duct like structures instead of capillary‐like networks, basing on the observation of epithelial surface markers CK 18 and CK‐19. However, because only 2D cultures were performed in our study, the observation of ductal structure was relatively limited. The 3D culture will be needed in future studies to further confirm the tube‐like structure formed by HBL‐100 induced ADSCs.

As cytokeratin 18, 19 are known to mainly exist in epithelial tissues, they are stably expressed in normal epithelial cells. Therefore, the observed positive expression of these two markers can indicate an epithelial differentiation 21, 22.

Recently autologous adipose tissue transplantation has been increasingly used clinically, especially for breast augmentation. It is well known that incidence rates of breast cancer have been increasing throughout the world 16 and adipose tissue is one of the richest sources of mesenchymal stem cells in the body 23. Our *in vitro* results may well indicate that the grafted ADSCs can form neoplasm‐like tissues in the breast and thus potentially increase the risk of breast carcinoma. These findings are consistent with our previous observation that a variety of cells such as endothelial progenitor cells, that can be found in aspirated fatty tissue, showed a high angiogenic potential and might facilitate neoangiogenesis and hence growth of tumour cells also in a human setting 24. The mechanism of the epithelial‐like differentiation of ADSCs is presently unknown. Recently, some researchers demonstrated that microRNA let‐7e induces the renal epithelial differentiation of ADSCs by down‐regulating the expression of matrix metalloproteinase‐9 25. More research is needed on the expression of microRNAs in ADSCs and HBL‐100 cells to clarify the mechanism.

Further studies should pay attention to the *in situ* culture of HBL‐100 induced ADSCs to provide more evidence, but our *in vitro* data suggest that further research with ADSC and mammary epithelial cell lines could be effective to elucidate a potential role of fat transplants in breast tissue and help to clarify safety issues that hitherto have not been sufficiently studied, despite an increasing number of clinical reports of transplanting aspirated fat into breasts.

Limitations of this study are that the phenotype of isolated ADSCs was not analysed; there may be differences in the potential of epithelial differentiation among subgroups of ADSCs. Secondly, no 3D cultures were performed, so that the tube formation of induced ADSCs needs future confirmation. Thirdly, at this stage, *in vitro* data give a first hint to the effects, but the growth of transplanted ADSCs needs to be further clarified after establishing an appropriate *in vivo* model.

## Conclusions

Adipose‐derived stem cells are not biological stable when co‐cultured with HBL‐100 cells. They differentiate into epithelial‐like cells with the expression of epithelial surface marks (CK 18, 19) and form tube‐like structures. This may offer an important evidence for the further study of clinical application of transplanting ADSCs rich adipose tissue into the breast in the future.

## Conflicts of interest

The authors confirm that there are no conflicts of interest.

## References

[1] Stewart CJ , Miller C , McPherson C , *et al* Patient's attitude towards the donation and use of adipose tissue and adipose derived stem cells for research. J Plast Reconstr Aesthet Surg. 2014; 68: 588–9. 25465768 10.1016/j.bjps.2014.10.049

[2] Horch RE , Beier JP , Kneser U , *et al* Successful human long‐term application of *in situ* bone tissue engineering. J Cell Mol Med. 2014; 18: 1478–85. 24801710 10.1111/jcmm.12296 PMC4124030

[3] Banyard DA , Salibian AA , Widgerow AD , *et al* Implications for human adipose‐derived stem cells in plastic surgery. J Cell Mol Med. 2014; 19: 21–30. 25425096 10.1111/jcmm.12425 PMC4288346

[4] Yoshimura K , Sato K , Aoi N , *et al* Cell‐assisted lipotransfer for cosmetic breast augmentation: supportive use of adipose‐derived stem/stromal cells. Aesthetic Plast Surg. 2008; 32: 48–55. 17763894 10.1007/s00266-007-9019-4 PMC2175019

[5] Gyorki DE , Asselin‐Labat M‐L , van Rooijen N , *et al* Resident macrophages influence stem cell activity in the mammary gland. Breast Cancer Res. 2009; 11: R62. 19706193 10.1186/bcr2353 PMC2750124

[6] Montales MTE , Rahal OM , Nakatani H , *et al* Repression of mammary adipogenesis by genistein limits mammosphere formation of human MCF‐7 cells. J Endocrinol. 2013; 218: 135–49. 23645249 10.1530/JOE-12-0520

[7] Bordonaro M , Lazarova DL . Hypothesis: cell signalling influences age‐related risk of colorectal cancer. J Cell Mol Med. 2014; 19: 74–81. 25388238 10.1111/jcmm.12366 PMC4288351

[8] Yang J , Xiong L , Wang R , *et al* Adipose‐derived stem cells from the breast. J Res Med Sci. 2014; 19: 112–6. 24778663 PMC3999595

[9] Xiong L , Sun J , Hirche C , *et al* *In vitro N*‐acetyl‐l‐cysteine promotes proliferation and suppresses interleukin‐8 expression in adipose‐derived stem cells. Aesthetic Plast Surg. 2012; 36: 1260–5. 22936379 10.1007/s00266-012-9960-8

[10] Zhong A , Wang G , Yang J , *et al* Stromal‐epithelial cell interactions and alteration of branching morphogenesis in macromastic mammary glands. J Cell Mol Med. 2014; 18: 1257–66. 24720804 10.1111/jcmm.12275 PMC4124011

[11] Wang L , Luo X , Lu Y , *et al* Is the resorption of grafted fat reduced in cell‐assisted lipotransfer for breast augmentation? Ann Plast Surg. 2015; 75: 128–34. 24691331 10.1097/SAP.0000000000000068

[12] Coleman SR , Saboeiro AP . Fat grafting to the breast revisited: safety and efficacy. Plast Reconstr Surg. 2007; 119: 775–85. 17312477 10.1097/01.prs.0000252001.59162.c9

[13] Delay E , Garson S , Tousson G , *et al* Fat injection to the breast: technique, results, and indications based on 880 procedures over 10 years. Aesthet Surg J. 2009; 29: 360–76. 19825464 10.1016/j.asj.2009.08.010

[14] Bielli A , Scioli MG , Gentile P , *et al* Adult adipose‐derived stem cells and breast cancer: a controversial relationship. Springerplus. 2014; 3: 345. 25089245 10.1186/2193-1801-3-345 PMC4117859

[15] Wang Y‐Y , Lehuédé C , Laurent V , *et al* Adipose tissue and breast epithelial cells: a dangerous dynamic duo in breast cancer. Cancer Lett. 2012; 324: 142–51. 22643115 10.1016/j.canlet.2012.05.019

[16] DeSantis C , Ma J , Bryan L , *et al* Breast cancer statistics, 2013. CA Cancer J Clin. 2013; 64: 52–62. 24114568 10.3322/caac.21203

[17] Sahar DE , Walker JA , Wang HT , *et al* Effect of endothelial differentiated adipose‐derived stem cells on vascularity and osteogenesis in poly(D, L‐lactide) scaffolds *in vivo* . J Craniofac Surg. 2012; 23: 913–8. 22627404 10.1097/SCS.0b013e31824e5cd8

[18] Eterno V , Zambelli A , Pavesi L , *et al* Adipose‐derived mesenchymal stem cells (ASCs) may favour breast cancer recurrence *via* HGF/c‐Met signaling. Oncotarget. 2013; 15: 613–33. 10.18632/oncotarget.1359 PMC3996669 24327602

[19] Strassburg S , Nienhueser H , Björn Stark G , *et al* Co‐culture of adipose‐derived stem cells and endothelial cells in fibrin induces angiogenesis and vasculogenesis in a chorioallantoic membrane model. J Tissue Eng Regen Med. 2013; doi: 10.1002/term.1769. 10.1002/term.1769 23712963

[20] Lozito TP , Tuan RS . Endothelial and cancer cells interact with mesenchymal stem cells *via* both microparticles and secreted factors. J Cell Mol Med. 2014; 18: 2372–84. 25250510 10.1111/jcmm.12391 PMC4302643

[21] Wan J‐X , Zou Z‐H , You D‐Y , *et al* Bone marrow‐derived mesenchymal stem cells differentiation into tubular epithelial‐like cells *in vitro* . Cell Biochem Funct. 2012; 30: 129–38. 22125055 10.1002/cbf.1826

[22] Vorotelyak EA , Cheremnykh ES , Vasil'ev AV , *et al* Expression of keratin 19 in a culture of human epidermal keratinocytes. Dokl Biol Sci. 2006; 408: 272–4. 16909997 10.1134/s0012496606030197

[23] Salinas HM , Broelsch GF , Fernandes JR , *et al* Comparative analysis of processing methods in fat grafting. Plast Reconstr Surg. 2014; 134: 675–83. 24945949 10.1097/PRS.0000000000000524

[24] Brandl A , Yuan Q , Boos AM , *et al* A novel early precursor cell population from rat bone marrow promotes angiogenesis *in vitro* . BMC Cell Biol. 2014; 15: 12. 24666638 10.1186/1471-2121-15-12 PMC3987126

[25] Ventayol M , Viñas JL , Sola A , *et al* miRNA let‐7e targeting MMP9 is involved in adipose‐derived stem cell differentiation toward epithelia. Cell Death Dis. 2014; 5: e1048. 24503540 10.1038/cddis.2014.2 PMC3944246

